# A dosimetric analysis of a rotating gamma ray system and volumetric modulated arc therapy in retrospective replanning for primary liver cancer

**DOI:** 10.1002/acm2.70581

**Published:** 2026-06-15

**Authors:** Jiarui Zhang, Siming Shi, Jingran Wu, Lei Shi, Liying Song, Meng Yang, Huiyang Yu, Peng Zhen, Fang Fang

**Affiliations:** ^1^ Department of Radiation Oncology, Chifeng Tumor Hospital Inner Mongolia Autonomous Region Chifeng China

**Keywords:** Gamma Knife, Primary Liver Cancer, VMAT

## Abstract

**Background:**

This study is a retrospective replanning study. For primary liver cancer, radiotherapy is vital for inoperable patients; however, dosimetric data comparing the rotating gamma ray system (RGS) and volumetric modulated arc therapy (VMAT) remains scarce, complicating clinical technique selection. Notably, RGS refers to a body gamma ray platform designed for extracranial stereotactic body radiotherapy (SBRT) — this is distinct from the “Gamma Knife” system, which is specifically dedicated to intracranial radiosurgery.

**Purpose:**

To compare the dosimetric differences between RGS and VMAT techniques in the radiotherapy of primary liver cancer.

**Methods:**

30 patients with primary liver cancer, underwent both RGS and VMAT planning. Analyzed planning target volume (PTV) parameters: coverage (Target Coverage Index, TCI), max/mean/min dose (D_max_/D_mean_/D_min_), conformity (Conformity Index, CI), homogeneity (Homogeneity Index, HI), gradient index (Gradient Index, GI). Evaluated liver doses: D_mean_, V_20_, V_5_.

**Results:**

In this dosimetric comparison of 30 primary liver cancer patients, RGS plans demonstrated significantly higher maximum tumor dose (D_max_: 10253 ± 196 cGy vs. VMAT: 6807 ± 315 cGy; *P* < 0.001), mean tumor dose (D_mean_ median [IQR]: RGS: 7410 cGy [7222–7493] vs. VMAT: 5780 cGy [5675–5870]; *P* < 0.001), and target coverage (median [IQR]: RGS: 100% [99–100] vs. VMAT: 98% [98–98]; *P* < 0.001) compared to VMAT plans. RGS also exhibited a steeper dose fall‐off (GI: 3.32 ± 0.37 vs. VMAT: 5.84 ± 1.12; *P* < 0.001). Conversely, VMAT achieved superior conformity (CI: 0.83 ± 0.06 vs. RGS: 0.63 ± 0.08; *P* < 0.001), homogeneity (HI: 0.23 ± 0.05 vs. RGS: 0.57 ± 0.05; *P* < 0.001), and significantly reduced low‐dose liver irradiation (V_5_: 23.7% ± 10.2% vs. RGS: 34.4% ± 12.8%; *P* < 0.001). The mean liver dose was also significantly lower with VMAT (616 ± 253 cGy vs. RGS: 677 ± 314 cGy; *P* = 0.039). No significant differences were observed in minimum tumor dose (D_min_) or liver V_20_.

**Conclusions:**

In radiotherapy for primary liver cancer, the rotating gamma ray system offers the characteristic of highly focused central dose with rapid peripheral dose fall‐off, ensuring high tumor dose while better protecting adjacent liver tissues. VMAT provides superior conformity and homogeneity, with better control of the mean liver dose and low‐dose irradiation volumes.

## 1 INTRODUCTION

Primary liver cancer, particularly hepatocellular carcinoma (HCC), is one of the leading causes of cancer‐related death globally, ranking fifth in incidence and second in mortality. It is estimated that approximately 750,000 people die from this disease annually.^1^ The pathogenesis of HCC is complex, closely associated with chronic liver disease, host factors, disease status, and environmental factors. Hepatitis B virus (HBV) and hepatitis C virus (HCV) infections are known major risk factors, and the geographical distribution of HCC highly coincides with the endemic areas of these viral hepatitis infections, especially in East Asia, Southeast Asia, and Central and Western Africa. Although surgical resection and liver transplantation are traditional treatments for HCC, many patients are diagnosed at an advanced stage and are ineligible for surgery. Therefore, developing effective non‐surgical treatment methods is crucial for these patients. In recent years, with advancements in medical technology, non‐surgical treatments such as transarterial chemoembolization (TACE), ablation therapy, radiotherapy, molecular targeted therapy, and biotherapy have shown significant therapeutic efficacy.^2^

The application of advanced techniques such as Intensity‐Modulated Radiation Therapy (IMRT) and Stereotactic Body Radiation Therapy (SBRT) has provided new possibilities for HCC treatment. These techniques enable precise delivery of high‐dose radiation to the tumor target while sparing surrounding normal liver tissue, thereby reducing the risk of radiation‐induced liver injury. Intensity‐Modulated Radiation Therapy (IMRT) and Volumetric Modulated Arc Therapy (VMAT) are two advanced radiotherapy techniques.^3^ Each has advantages in dose distribution, treatment efficiency, and normal tissue protection, offering more treatment options for liver cancer patients. Studies have found that although both techniques can provide satisfactory dose distributions, VMAT offers shorter treatment times, indicating its advantage in improving treatment efficiency.^4^ Another study noted that VMAT is slightly superior to IMRT in terms of dose conformity and homogeneity, especially for tumors with complex shapes or indistinct boundaries with surrounding tissues.^5^

The Rotating Gamma ray System (RGS) is a dedicated stereotactic radiotherapy device. It employs an array of 30 Cobalt‐60 (Co‐60) sources which rotate concentrically as a single unit about a common axis perpendicular to the patient. This motion converges highly collimated beams onto the target, creating a highly conformal dose distribution. This unique delivery mechanism, combined with variable collimation, offers distinct technical advantages for treating both intracranial and extracranial indications.^6^ VMAT provides highly conformal dose distributions and higher treatment efficiency through continuous dynamic beam modulation.^7^ However, dosimetric studies comparing RGS and VMAT for primary liver cancer are relatively limited in literature. This study aims to evaluate the dosimetric characteristics and differences in liver protection between these two radiotherapy techniques through comparative analysis.

## 2 RESEARCH METHODS

### 2.1 Patient data

Thirty patients with primary liver cancer treated in the Department of Radiation Oncology at Chifeng Tumor Hospital, between January 2023 and May 2025 were selected. Inclusion criteria: Age between 18 and 75 years, any gender; clinically or pathologically confirmed primary liver cancer; patients unsuitable for surgery or explicitly refusing surgical treatment; single lesion with diameter ≤ 5 cm; Eastern Cooperative Oncology Group Performance Status (ECOG PS)^8^ score of 0 or 1; Child‐Pugh liver function classification^9^ grade A or B; absence of hepatic encephalopathy and/or moderate or large ascites. Exclusion criteria: History of esophageal variceal bleeding, severe hypersplenism syndrome; refractory ascites; previous history of liver radiotherapy; metastatic cancer to other sites; PS score > 1; Child‐Pugh grade C; other severe underlying diseases. Comprehensive clinical evaluation and auxiliary examinations were performed according to the American Joint Committee on Cancer (AJCC) Liver Cancer Staging System (8th edition)^10^ to confirm patient eligibility. This retrospective study complied with the Declaration of Helsinki and was approved by the Ethics Review Committee of Chifeng Tumor Hospital, (Approval No.: 2024 New No. 73); informed consent was waived for the retrospective study.

### 2.2 Positioning and CT scanning

Patients were positioned supine with arms raised, crossed and elbows resting on the forehead, and immobilized using a vacuum bag or thermoplastic film with an abdominal belt applied for respiratory motion reduction. Four‐dimensional computed tomography (4D‐CT) was performed to acquire volumetric images across the entire respiratory phase using a GE 16‐slice spiral CT scanner with intravenous contrast enhancement; the scan slice thickness was set at 2.5 mm. All acquired images were subsequently transferred to the Elekta linear accelerator treatment planning system (Monaco 5.11.0, Elekta, Sweden) for further processing.

### 2.3 Target volume delineation

According to the International Commission on Radiation Units and Measurements (ICRU) Reports 50 and 83,^11^, ^12^ the target volumes and organs at risk (OARs) were manually delineated by radiation oncologists and medical physicists on the planning CT images, with reference to diagnostic CT and MRI. The gross tumor volume (GTV) was defined based on the visible extent of the solid tumor. An internal target volume (ITV) was then created to encompass the GTV motion throughout the respiratory cycle. Finally, a planning target volume (PTV) was generated by adding a margin to the ITV to account for setup uncertainties and residual positional errors.

### 2.4 Treatment planning

CT images, together with delineated target volumes and OAR contours from all 30 patients, were imported into two independent treatment planning systems: RGS planning system (OUR‐BTPS2 1.0, OUR United Xi'an, China) and the linear accelerator–based treatment planning system (Monaco 5.11.0, Elekta, Sweden).

### 2.5 RGS plan

The rotating gamma ray system plans^13^ were designed using a multi‐source γ‐ray stereotactic radiotherapy system (OUR United Xi'an, China), which incorporates 30 sealed ^60^Co sources and three sets of collimators (diameters: 1, 3, and 5 cm) at a fixed source‐to‐focus distance of 45 cm. Dose calculation was executed using a fast photon dose algorithm that supports tissue density heterogeneity correction based on CT values, thereby facilitating a more precise quantification of the radiation dose distribution within distinct tissue types. A co‐planar rotational technique was employed, with the treatment couch moving sequentially to predefined coordinates. Gantry angles were pre‐optimized and validated through collision simulations to ensure safe delivery. The primary dosimetric objective mandated coverage of ≥ 95% of the PTV by the 50% isodose line. Plan optimization further aimed to enhance dose conformality and reduce treatment time through optimal collimator selection and beam angle fine‐tuning.

### 2.6 VMAT plan

VMAT plans were generated on a Versa‐HD linear accelerator (Elekta, Sweden) equipped with an Agility 80‐leaf MLC (5 mm leaf width at isocenter) using a 6 MV X‐ray beam at a maximum dose rate of 600 MU/min. The Monte Carlo algorithm was used for dose calculation. Plans utilized 1–2 coplanar arcs, with their angles and quantity optimized based on tumor geometry and proximity to OARs. The dosimetric criteria required that ≥ 95% of the PTV receive 100% of the prescription dose, while the D_max_ to the PTV was constrained to < 150%. Care was taken to eliminate cold spots within the GTV and avoid hot spots outside the PTV. The optimization priority was to minimize OAR doses without compromising target coverage.

### 2.7 Dosimetric evaluation

Plans were compared using dose distribution maps (axial, coronal, sagittal planes) and dose‐volume histograms (DVHs) for the PTV and OARs. Dosimetric parameters collected for both plans included: PTV maximum dose (D_max_), mean dose (D_mean_), minimum dose (D_min_), target coverage index (TCI), conformity index (CI), homogeneity index (HI), and gradient index (GI),^14^ calculated as follows:

$$\mathrm{TCI}=\frac{TV_{\mathrm{RI}}}{TV}$$

$$\mathrm{CI}=\frac{{\left(TV_{\mathrm{RI}}\right)}^{2}}{TV\times V_{\mathrm{RI}}}$$

$$\mathrm{HI}=\frac{D_{2\%}-D_{98\%}}{D_{50\%}}$$

$$\mathrm{GI}=\frac{PIV_{50\%}}{PIV}$$

where TV_RI_ is the target volume receiving the prescription dose or higher, TV is the target (PTV) volume, V_RI_ is the total volume covered by the prescription isodose line, D_2%_ is the dose received by 2% of the PTV volume,D_98%_ is the dose received by 98% of the PTV volume, D_50%_ is the dose received by 50% of the PTV volume (median dose), PIV is the prescription isodose volume, and PIV_50%_ is the 50% prescription isodose volume. CI ranges from 0 to 1; values closer to 1 indicate better conformity. Lower HI values indicate more homogeneous dose distribution. Lower GI values indicate steeper dose fall‐off. Additionally, the mean liver dose (D_mean_), the percentage volume of liver receiving ≥ 20 Gy (V_20_), and the percentage volume receiving ≥ 5 Gy (V_5_) were recorded.

### 2.8 Statistical analysis

Data were analyzed using IBM SPSS Statistics 27.0 (IBM Corp., Armonk, NY, USA). Categorical data were described using frequency and percentage. Normality was assessed using the Shapiro–Wilk test. Normally distributed data were described using mean ± standard deviation $\left(\bar{x}\pm s\right)$, while non‐normally distributed data were described using median (interquartile range, M [*P*_25_, *P*_75_]). Differences between RGS and VMAT plan parameters were analyzed using paired‐sample *t*‐tests (normally distributed data) or Wilcoxon signed‐rank tests (non‐normally distributed data). The significance level was set at *α* = 0.05, with *P* < 0.05 considered statistically significant.

## 3 RESULTS

A total of 30 primary liver cancer patients were enrolled. Lesion locations: Segment II (*n* = 2, 6.7%); Segment III (*n* = 1, 3.3%); Segment IV(*n* = 11, 36.7%); Segment V (*n* = 5, 16.7%); Segment VII (*n* = 3, 10.0%); Segment VIII(*n* = 8, 26.7%). Fraction dose: 400 cGy (*n* = 19, 63.3%); 500 cGy (*n* = 11, 36.7%). Number of fractions: 10 (*n* = 11, 36.7%); 13 (*n* = 19, 63.3%). Total prescription dose: 5000 cGy (*n* = 11, 36.7%); 5200 cGy (*n* = 19, 63.3%). These regimens align with established SBRT conventions for primary liver cancer, where total doses of 48–60 Gy are commonly recommended, yielding a biologically effective dose (BED) range of 71.4–137.7 Gy when calculated using an *α/β* ratio of 10 Gy.^15^, ^16^ Details are shown in Table 1.

**TABLE 1:** Basic characteristics of enrolled patients (*n* = 30).

Item	Category	Number (*n*)	Percentage (%)
Lesion location	Segment II	2	6.7
	Segment III	1	3.3
	Segment IV	11	36.7
	Segment V	5	16.7
	Segment VII	3	10.0
	Segment VIII	8	26.7
Fraction dose (cGy)	400	19	63.3
	500	11	36.7
Number of fractions (*f*)	10	11	36.7
	13	19	63.3
Prescription dose (cGy)	5000	11	36.7
	5200	19	63.3

### 3.1 Target volume details

GTV ranged from 1.02 to 54.78 cm^3^ (median 12.44 cm^3^); PTV ranged from 7.16 to 110.27 cm^3^ (median 33.14 cm^3^); PTV diameter ranged from 2.4 to 5.9 cm (median 3.90 cm); Liver volume ranged from 1028.62 to 2227.40 cm^3^ (median 1395.41 cm^3^). Details are shown in Table 2.

**TABLE 2:** Target volume characteristics (*n* = 30).

Item	Min	Max	M(P25,P75)
GTV Volume (cm^3^)	1.02	54.78	12.44(4.30,19.98)
PTV Volume (cm^3^)	7.16	110.27	33.14(18.51,48.45)
PTV Diameter (cm)	2.4	5.9	3.90(3.58,4.83)
Liver Volume (cm^3^)	1028.62	2227.40	1395.41(1244.00,1734.01)

**FIGURE 1:**
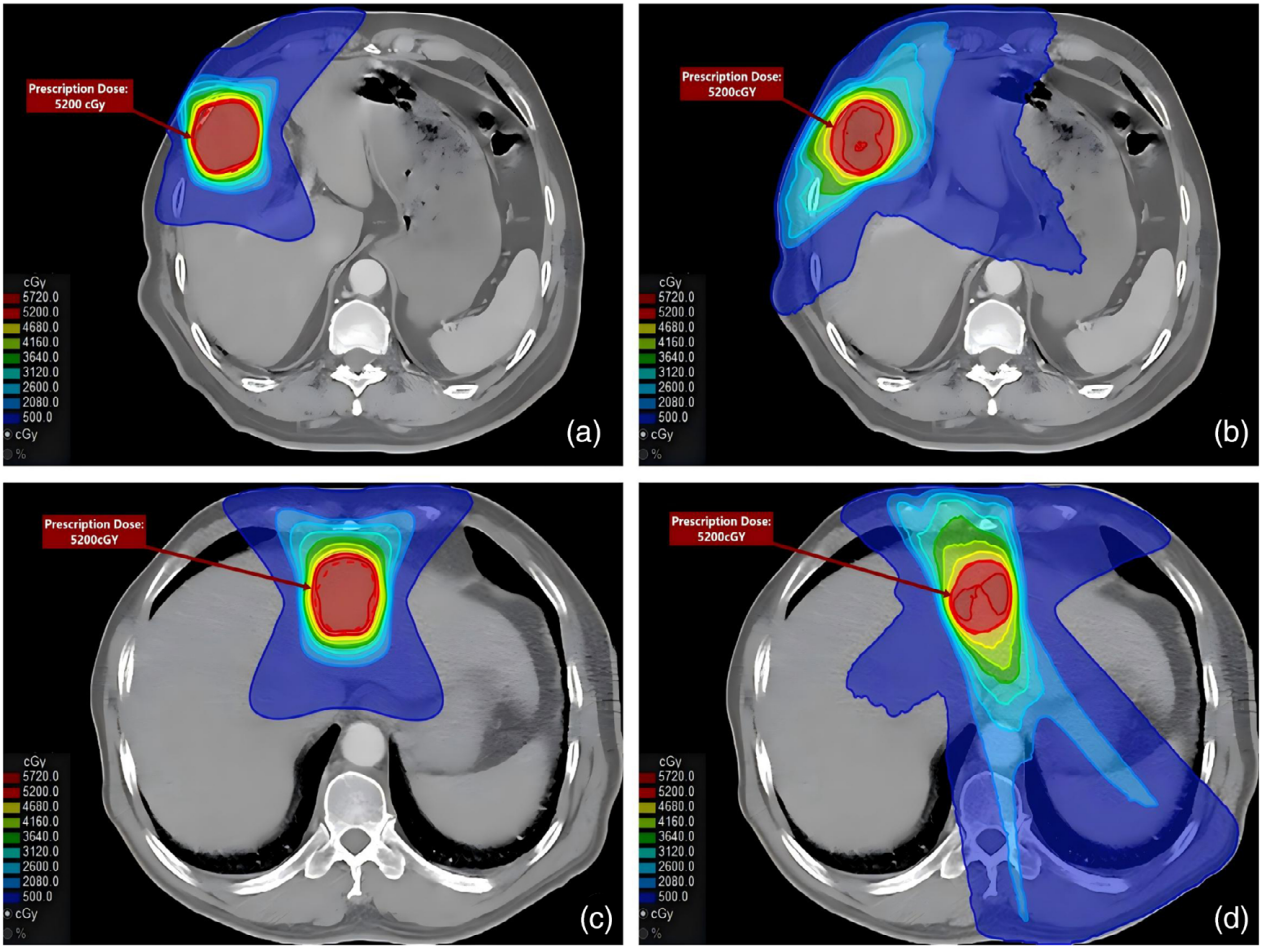
This figure illustrates the isodose distributions for both techniques in two representative patients. (a) RGS plan for Patient 1; (b) VMAT plan for Patient 1; (c) RGS plan for Patient 2; (d) VMAT plan for Patient 2.

After evaluation by no less than two associate chief physicians and at least one senior physicist, a total of 60 radiotherapy plans (30 RGS, 30 VMAT) were deemed clinically acceptable. No patient was encountered for whom either planning system failed to generate a clinically acceptable plan. Plan parameters were evaluated using dose‐volume histograms (DVHs). Figure 1 illustrates the isodose distributions for both techniques in two representative. Significant differences in dosimetric parameters between RGS and VMAT plans were observed, as presented in Table 3.

**TABLE 3:** Detailed plan comparison (*n* = 30).

Item	RGS	VMAT	t/Z	*P*
	$\bar{x}\pm s$ /M (P25, P75)	$\bar{x}\pm s$ /M (P25, P75)		
D_max_ (cGy)†	10253.33 ± 196.05	6806.77 ± 315.12	49.317	< 0.001
D_min_ (cGy)^a^	4640.50 (4518.25, 4817.50)	4750.00 (4454.25, 4942.25)	−0.031^b^	0.975
D_mean_ (cGy)^a^	7410.00 (7222.25, 7492.50)	5779.50 (5675.25, 5870.00)	−4.782^c^	< 0.001
TCI (%)^a^	100 (99, 100)	98 (98, 98)	−4.689^c^	<0.001
PIV (cm^3^)^a^	55.23 (30.22, 69.52)	38.175 (21.21, 55.9225)	−4.782^c^	< 0.001
PIV_50%_ (cm^3^)†	189.79 ± 115.68	228.617 ± 115.51178	−4.85	< 0.001
CI†	0.6257 ± 0.0786	0.8267 ± 0.0576	−13.163	< 0.001
GI†	3.3230 ± 0.3688	5.8373 ± 1.1167	−13.285	< 0.001
HI†	0.5730 ± 0.0500	0.2298 ± 0.0451	24.954	< 0.001
Liver_V_5_ (%)†	34.43 ± 12.78	23.67 ± 10.19	6.75	< 0.001
Liver_V_20_ (%)^a^	10 (5.75, 14.5)	10 (6, 13.25)	−0.312^b^	0.755
D_mean_ †	677.35 ± 314.26	616.23 ± 252.85	2.157	0.039

^†^ Paired‐sample *t‐test*.

^a^ Wilcoxon test.

^b^ Based on positive ranks.

^c^ Based on negative ranks.

For the PTV: RGS plans had significantly higher maximum dose (D_max_: 10253.33 ± 196.05 cGy vs. VMAT: 6806.77 ± 315.12 cGy, *p* < 0.001) and mean dose (D_mean_ Median [IQR]: RGS: 7410.00 cGy [7222.25–7492.50] vs.VMAT: 5779.50 cGy [5675.25–5870.00], *P* < 0.001). There was no significant difference in minimum dose (D_min_, *P* = 0.975). RGS showed superior target coverage (Median: 100% [99–100] vs. VMAT: 98% [98–98], *P* < 0.001). Dose‐volume parameters showed that RGS had a significantly larger prescription isodose volume (PIV Median [IQR]: 55.23 cm^3^ [30.22–69.52] vs. VMAT: 38.175 cm^3^ [21.21–55.9225], *P* < 0.001), but a significantly smaller 50% prescription isodose volume (PIV_50%_: 189.79 ± 115.68 cm^3^ vs. VMAT: 228.62 ± 115.51 cm^3^, *P* < 0.001).

Plan quality indices showed VMAT had superior conformity index (CI: 0.8267 ± 0.0576 vs. RGS: 0.6257 ± 0.0786, *P* < 0.001) and homogeneity index (HI: 0.2298 ± 0.0451 vs. RGS: 0.5730 ± 0.0500, *P* < 0.001), while RGS had a significantly better gradient index (GI: 3.3230 ± 0.3688 vs. VMAT: 5.8373 ± 1.1167, *P* < 0.001).

For normal liver protection, RGS resulted in a significantly higher mean liver dose (D_mean_: 677.35 ± 314.26 cGy vs. VMAT: 616.23 ± 252.85 cGy, *P* = 0.039) and liver V_5_ (34.43 ± 12.78% vs. VMAT: 23.67 ± 10.19%, *P* < 0.001). There was no significant difference in liver V_20_ (Median: RGS 10% [5.75, 14.50], VMAT 10% [6.00, 13.25], *P* = 0.755).

## 4 DISCUSSION

To date, our institution has utilized RGS to treat more than 120 patients with primary liver cancer. Encouragingly, favorable clinical outcomes have been observed, with no Grade ≥ 3 acute adverse events reported in any of these patients. While long‐term follow‐up data are still being systematically collected and analyzed, these preliminary findings strongly support the favorable safety profile and promising efficacy of RGS in this clinical setting. Further prospective studies with extended follow‐up durations are warranted to confirm these results and comprehensively evaluate long‐term oncologic outcomes, including local control and survival.

Our study demonstrates that both RGS and VMAT can meet clinical requirements for radiotherapy in primary liver cancer. The two techniques exhibit differences in some parameters related to target dose indices, plan quality indices, and protection of normal liver tissue. Dosimetric analysis revealed that RGS plans delivered significantly higher maximum dose (D_max_) and mean dose (D_mean_) to the PTV compared to VMAT plans, while no statistically significant difference was observed in the minimum dose (D_min_) to the PTV. This result is consistent with the study by Duan et al.,^17^ indicating that both RGS and VMAT ensure the minimum target dose meets clinical standards, effectively achieving the goal of treating primary liver cancer. Furthermore, RGS delivers higher overall dose levels and can provide higher hotspot doses, which offers a therapeutic advantage for lesions requiring high‐dose irradiation for tumor kill, especially for radioresistant tumors. However, in practical application, careful attention must be paid to the location of hotspot doses to avoid potential radiation‐induced injuries, ensuring treatment safety and efficacy.

RGS plans achieved higher target coverage and larger prescription isodose volumes than VMAT plans. The results show that RGS completely covered the target (100%), while VMAT showed slight under‐coverage (98%). Conversely, the 50% prescription isodose volume was smaller for RGS than for VMAT. The rotating gamma ray system utilizes 30 fixed Co‐60 sources for highly focused irradiation, creating spherical dose distributions through multi‐isocenter superposition. Its dose fall‐off is extremely steep, achieving nearly vertical dose attenuation at the target edge.^18^ This characteristic allows it to strictly confine the high‐dose region within the target, facilitating 100% coverage while maximizing protection of surrounding tissues. VMAT irradiation with a linear accelerator, modulated by a multileaf collimator (MLC) and optimized using inverse planning algorithms, requires trade‐offs between target coverage, conformity, and normal tissue sparing. It may intentionally allow slight under‐coverage at the target edge to reduce low‐dose exposure to normal organs.^19^ Nevertheless, 98% coverage still meets the ICRU‐91 report^20^ requirement that ≥ 95% of the PTV volume receives the prescription dose.

Comparing the conformity index (CI) and homogeneity index (HI) between VMAT and RGS plans, VMAT demonstrated superior performance in both conformity and homogeneity, consistent with the findings of Cao et al.^21^ This indicates that the prescription dose distribution in VMAT plans conforms more closely to the target shape, and the dose within the target is more uniform. The gradient index (GI) of RGS plans was significantly better than that of VMAT plans. A lower GI value signifies a steeper dose fall‐off from high to low, aiding in the precise distinction between high target dose and low dose to adjacent normal tissues. The significantly lower GI value for RGS constitutes its core advantage. This result further confirms the superiority of RGS plans in terms of the volume covered by the 50% prescription dose, indicating its ability to minimize the risk of intermediate‐to‐high dose exposure to normal tissues outside the target.

Regarding liver dose, the results showed that liver V_5_ and mean radiation dose (D_mean_) were significantly higher in RGS plans than in VMAT plans, while there was no statistical difference in liver V_20_ between the two techniques. This suggests that although RGS reduces intermediate‐to‐high dose exposure to normal tissues through rapid dose fall‐off, it offers less protection against low‐dose irradiation to the liver compared to VMAT.Studies have demonstrated the clinical relevance of these low‐dose liver metrics. Specifically, in patients with Child‐Pugh A cirrhosis undergoing hypofractionated conformal radiotherapy for primary liver cancer, all V_5_–V_40_ parameters were significantly higher in those who developed classic radiation‐induced liver disease (RILD) compared to non‐RILD patients (all *p* < 0.01), indicating that low‐dose irradiation volumes may be associated with increased RILD risk and thus require careful clinical control.^22^ Furthermore, a separate study in hepatocellular carcinoma patients treated with intensity‐modulated radiotherapy identified a mean liver dose ≥ 13.7 Gy as a significant predictor of non‐classic RILD (ncRILD).^23^ Collectively, these findings underscore that our observed dosimetric differences—higher V_5_ and D_mean_ with RGS—may carry clinical implications for radiation‐induced hepatic toxicity, particularly in patients with underlying liver dysfunction.

## 5 CONCLUSIONS

Both RGS and VMAT can meet clinical requirements for the treatment of primary liver cancer. The rotating gamma ray system, with its ultra‐high dose concentration and steep gradient, offers maximum protection to critical structures. It holds considerable advantages for treating small, regularly shaped, or radioresistant lesions. However, vigilance is required regarding the potential risks associated with its low‐dose bath to the liver. VMAT plans, with their superior conformity and target homogeneity, perform better in controlling lowdose irradiation volumes. They are more advantageous for treating complex or irregularly shaped, larger lesions. However, for small lesions adjacent to critical organs, the protection of normal tissues from intermediate‐to‐high doses may be less precise than with RGS. Therefore, in clinical practice for primary liver cancer, the choice of radiotherapy technique should be based on a comprehensive assessment of factors including lesion size, shape, location, treatment goal (curative or palliative), patient life expectancy, and tolerance to potential side effects associated with different dose levels.

Notably, while RGS systems are not widely available globally, the dosimetric advantages demonstrated in this study—particularly the steep dose gradient and reduced low‐dose exposure to normal liver tissue—provide valuable insights for optimizing SBRT strategies for small‐volume primary liver cancer, even in settings where more commonly used linac‐based VMAT systems or CyberKnife platforms are the primary treatment modalities. The dosimetric principles identified here, such as prioritizing steep dose falloff to minimize hepatic low‐dose exposure, are broadly applicable to SBRT planning across different technologies. Moving forward, our team will conduct expanded clinical investigations to further validate the long‐term efficacy and safety profiles of RGS, including comprehensive assessments of treatment‐related adverse events and oncologic outcomes in a larger, more diverse patient cohort.

## AUTHOR CONTRIBUTIONS

Jiarui Zhang and Siming Shi, as co‐first authors, made equal contributions to this study, including participating in study design, conducting data analysis, and drafting the manuscript; Jingran Wu, Lei Shi, Liying Song, Meng Yang, and Huiyang Yu were responsible for data collection, implementation of treatment plans, and proofreading of the manuscript; Peng Zhen and Fang Fang, serving as corresponding authors, supervised the entire study, revised the manuscript, and approved the final version for submission.

## CONFLICT OF INTEREST STATEMENT

The authors declare no conflicts of interest.

## ETHICS STATEMENT

This retrospective study complied with the Declaration of Helsinki and was approved by the Ethics Review Committee of Chifeng Tumor Hospital, (Approval No.: 2024 New No. 73); informed consent was waived for the retrospective study.

## Data Availability

Research data are stored in an institutional repository and will be shared upon request to the corresponding author.
